# Determinants and Motivations of Vaccination Hesitancy and Uptake in Nurses: A Systematic Review and Meta‐Analysis

**DOI:** 10.1111/jocn.17852

**Published:** 2025-06-25

**Authors:** Giulia Locatelli, Michela Luciani, Diletta Fabrizi, Beatrice Albanesi, Alessio Conti, Marco Clari, Erika Renzi, Azzurra Massimi, Davide Ausili

**Affiliations:** ^1^ School of Medicine and Surgery University of Milano‐Bicocca Monza Italy; ^2^ Department of Sciences of Public Health and Pediatrics University of Torino Torino Italy; ^3^ Department of Public Health and Infectious Diseases Sapienza University of Rome Rome Italy

**Keywords:** motivation, nurses, systematic review, vaccination hesitancy, vaccination uptake, vaccines

## Abstract

**Aim:**

Vaccinations are essential to ensure protection for healthcare professionals, patients and communities. However, vaccination hesitancy has been reported among healthcare professionals. Nurses are the main, first and direct point of contact for patients and citizens in most healthcare services, but only a minority of studies investigated vaccination hesitancy and uptake specifically in this population. Thus, this study aimed to explore the determinants and motivations of vaccination hesitancy and uptake among nurses.

**Design:**

Systematic review with a narrative synthesis approach.

**Methods:**

We included primary research exploring determinants or motivations of vaccination hesitancy or uptake among nurses. No time or geographical limit was applied. Generalised random‐effects linear models with a logit link were used to calculate the pooled estimated proportions for vaccine uptake among nurses.

**Data Sources:**

We explored nine databases (2023).

**Results:**

The initial search identified 3452 records; 42 records were eventually included in this review. Older age, longer professional experience, lack of confidence in vaccine safety and effectiveness and cost associated with the vaccine were among the most common determinants of vaccine hesitancy. Safety concerns, complacency (e.g., beliefs of not needing the vaccine), and accessibility (e.g., logistics) were among the most common motivations for being vaccine hesitant. Having strong confidence in the vaccine, a high sense of collective responsibility, previous vaccination uptake/positive intentions towards future vaccination, weaker vaccine complacency, and older age were among the most common determinants of vaccine uptake. Willingness to protect themselves and/or others, contribute to the herd immunity, and comply with recommendations were among the most common motivations for vaccine uptake. The pooled prevalence of influenza vaccine uptake among nurses was 44% (95% CI: 35–73).

**Conclusion:**

The findings of this systematic review with meta‐analysis could guide the identification of strategies to reduce barriers and further improve facilitators to eventually increase vaccine uptake in nurses.

**Implications for the Profession and/or Patient Care:**

This study contributes to further understanding nurses' beliefs, barriers, and facilitators towards vaccination. By doing so, these results could guide the identification of strategies to reduce barriers and further improve facilitators to eventually increase vaccine uptake in nurses.

**Reporting Method:**

We have adhered to relevant EQUATOR guidelines, in particular to the PRISMA checklist.

**Patient or Public Contribution:**

No patient or public contribution.

**Trial Registration:**

PROSPERO number: CRD42020212252

**Summary**

- Impact
  ○ This systematic review aimed to explore the determinants and motivations of vaccination hesitancy and uptake among nurses.
  ○ We found several variables being common determinants of vaccine hesitancy in nurses (e.g., older age, longer professional experience, lack of confidence in vaccine effectiveness, and cost associated with the vaccine), motivations for being vaccine hesitant (e.g., lack of confidence in vaccine safety, beliefs of not needing the vaccine, and logistics issues), determinants of vaccine uptake (e.g., having strong confidence in the vaccine, a high sense of collective responsibility, previous vaccination uptake/positive intentions towards future vaccination, and weaker vaccine complacency), and motivations for vaccine uptake (e.g., willingness to protect themselves and/or others, contribute to the heard immunity, and comply with recommendations).
  ○ These results contribute to further understanding the beliefs, barriers, and facilitators among nurses towards vaccination and, therefore, can guide the identification of strategies to reduce the barriers and further improve the facilitators to eventually increase vaccine uptake in nurses.
- What does this paper contribute to the wider global clinical community?
  ○ There are several variables acting as determinants and motivators for vaccine hesitancy and uptake in nurses.
  ○ Knowing such determinants and motivators can guide the identification of strategies to reduce the barriers and further improve facilitators to eventually increase vaccine uptake in nurses.

## 1 Introduction

Through vaccination, immunity increases in a population and, in this way, the spread of an infectious disease is limited by the reduction of susceptible hosts. Thus, maintaining herd immunity is crucial to control the spread of communicable diseases over the long term (Ashby and Best 2021). Besides herd immunity in the general population, it is of extreme importance that healthcare professionals are fully vaccinated because of the higher risk of exposure and transmission of some communicable diseases in healthcare settings compared to the general population. In addition, healthcare professionals could contribute to nosocomial transmission of diseases to vulnerable patients. Thus, protection of healthcare professionals through vaccination is essential to ensure protection for themselves, their patients, and communities (World Health Organization 2022a).

Based on the WHO's framework (World Health Organization 2022b) of behaviours and social drivers of vaccination, social processes (e.g., support of families, health worker recommendations) and ‘thinking and feeling’ factors (e.g., perceived disease risk and confidence in the vaccine) influence the motivation of an individual to get vaccinated. Once the motivation is set, practical issues (e.g., availability, affordability) will further influence if the person will actually and eventually receive the vaccination or not. The percentages of immunisation coverage globally range between 45% (birth dose of Hepatitis B and Yellow fever vaccine) to 89% (diphtheria, pertussis, and tetanus vaccines) (World Health Organization 2025). Besides practical issues that can influence the chance of receiving a vaccine, particularly in disadvantaged countries, social and individual processes also play a role in influencing the decision of getting vaccinated (World Health Organization 2022b). Vaccine hesitancy is defined as a state of indecisiveness regarding a vaccination decision (Larson 2022), or, also, as a delay in acceptance or refusal of vaccination (MacDonald 2015). Among the determinants for vaccine hesitancy there are contextual factors, individual and group factors, and vaccine‐specific issues as described by the SAGE Working Group on Vaccine Hesitancy (2014). Contextual factors refer to historic, socio‐cultural, environmental, institutional, economic, or political factors and they include communication and media environments, historical and programmes influences, policies, geographical barriers, and perceptions of the pharmaceutical industry (SAGE Working Group on Vaccine Hesitancy 2014). Individual and group factors include personal and family members' experience with vaccination, beliefs about health and prevention, medical knowledge, trust in the health system, perceived risk/benefit, and perceptions about immunisation as a social norm versus not needed/harmful (SAGE Working Group on Vaccine Hesitancy 2014). Lastly, vaccine‐specific issues include the scientific evidence of risk/benefit, the mode of administration and delivery, the vaccination schedule, costs, and the strength of recommendation (SAGE Working Group on Vaccine Hesitancy 2014).

The SAGE Working Group also elaborated a Confidence, Complacency, Convenience (3C) Model of Vaccine hesitancy (SAGE Working Group on Vaccine Hesitancy 2014) describing that vaccine hesitancy is a behaviour that reflects a constellation of factors and interactions between constructs. In particular, this model explains that vaccine hesitancy may result from the intersection between Confidence (i.e., trust in the effectiveness and safety of vaccines, in the system that delivers them, and in the motivations of the policymakers on the vaccines), Complacency (i.e., low perceived risk of vaccine‐preventable diseases and vaccination is not deemed a necessary preventive action), and Convenience (i.e., extent to which availability, affordability, and willingness‐to‐pay affect vaccine uptake).

### 1.1 The Review

The phenomenon of vaccination hesitancy is not only limited to the general population, but it also exists among healthcare professionals. Studies reported heterogeneous vaccination hesitancy rates among healthcare professionals and vaccination uptake among healthcare professionals has been reported lower than desirable (Lytras et al. 2016; Genovese et al. 2019). However, only a minority of studies investigated vaccination intentions specifically among nurses, who are the main, first, and direct point of contact for patients and citizens in most healthcare services (Flaubert et al. 2021; Sundler et al. 2023).

To be able to face and manage the phenomenon of vaccine hesitancy, it is pivotal to have a synthesis of the literature to understand which are the beliefs, barriers, and facilitators among nurses towards vaccination, which is currently lacking. Knowing the barriers and facilitators towards vaccination among nurses could guide the identification of strategies to reduce such barriers and further improve facilitators to eventually increase vaccine uptake.

### 1.2 Aim

In this systematic review and meta‐analysis, we aimed to explore the determinants and motivations of vaccination hesitancy and uptake among nurses.

## 2 Methods

### 2.1 Design

Systematic review with a narrative synthesis approach (Aromataris and Munn 2020) to identify the determinants and motivations of both vaccine hesitancy and uptake among nurses.

Motivation is defined as the energising of behaviour in pursuit of a goal (Simpson and Balsam 2016). Thus, in that regard, we will consider as motivators the elements that nurses in the included studies refer to influencing their decision to accept or avoid vaccines.

A determinant is a factor that makes something happen or leads to an outcome and implies some level of causation (Hahn 2021). Thus, in this review, we will consider as determinants the elements that have been statistically analysed and shown as influencing the outcome of accepting or avoiding vaccines.

### 2.2 Search Methods

We searched PubMed, Scopus, Psychinfo, Embase, CINAHL, Eric, Joanna Briggs, Cochrane database, and Web of Science. The main search terms included: (vaccine OR vaccination) AND (hesitancy OR resistance OR reluctance OR acceptance OR willingness OR uptake) AND (barrier OR determinant OR predictor OR intention) AND (nurse). MeSH terms have been used when appropriate. More details on the search strategies and search outcomes can be found in Appendix S1. Searches were performed in February 2023.

### 2.3 Inclusion and Exclusion Criteria

See Table 1.

**TABLE 1:** Inclusion and exclusion criteria.

Inclusion criteria	All primary research: – Including nurses – Investigating vaccine hesitancy or uptake – Investigating barriers or predictors of vaccine hesitancy or uptake
Exclusion criteria	Non‐primary research (e.g., literature reviews), including case studies, protocols and abstracts: – Including animals – Exclusively including students or healthcare professions other than nurses – Including students or healthcare professions other than nurses, without differentiating the outcomes specific for nurses only – Exclusively investigating COVID‐vaccine – Investigating COVID‐vaccine, without differentiating the outcomes regarding the other types of investigated vaccine

### 2.4 Data Abstraction and Synthesis

**FIGURE 1:**
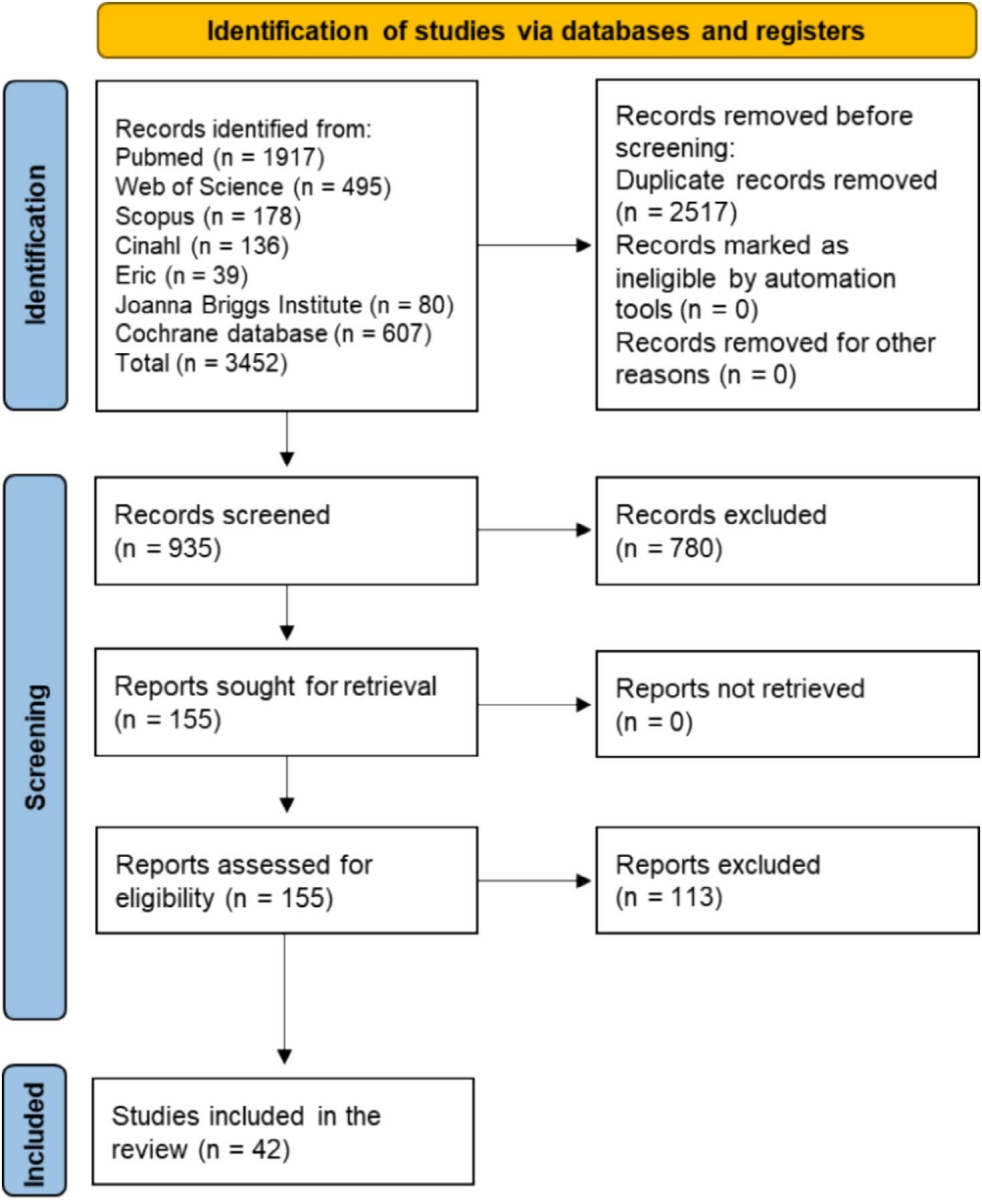
PRISMA flow diagram. [Colour figure can be viewed at wileyonlinelibrary.com]

The identified records were uploaded into Zotero and then into Rayyan web application (Ouzzani et al. 2016) to remove duplicates and conduct title and abstract screening. Then, two reviewers (G.L. and M.L.) independently screened the articles titles and abstracts to identify those that met inclusion criteria. Articles were labelled by each independent reviewer as ‘include for full‐text screening’, ‘exclude’, or ‘maybe keep the record’. At the end of this phase, the same two reviewers discussed and solved minor discrepancies. Afterwards, the same two reviewers screened the full texts of the remaining articles through the same process (include/exclude/maybe) of the first phase. The data extraction process is documented using the PRISMA 2020 flow diagram (Page et al. 2021) shown in Figure 1, and inter‐rater reliability is reported in Section 3. For each included study, data were extracted through the web application Joanna Briggs Institute SUMARI (Munn et al. 2019) using the relevant data extraction tool. Data extracted included the country and setting, study design, sample and sample size, instruments for data collection, vaccine type, vaccine uptake, determinants and motivations of vaccine hesitancy, and/or determinants and motivations of vaccine uptake.

Given the aim of this systematic review, we included any study design, any population, and vaccine type meeting our inclusion criteria. Data have been synthesised using a descriptive narrative approach, which summarises the evidence extracted from the included studies in words.

### 2.5 Statistical Analysis

Generalised random‐effects linear models with a logit link were used to calculate the pooled estimated proportions for vaccine acceptance and uptake among nurse populations (Lin and Chu 2020). Proportion estimates of vaccine intentions and coverage were included in the meta‐analysis when more than five estimates were available for a given vaccine. Additionally, to grant homogeneity in the analysis, we only considered studies referring to the influenza vaccination, which were the vast majority, because for other vaccines (i.e., HBV and HPV) the number of eligible studies was too limited or the available data too heterogeneous to support a reliable quantitative synthesis. Only proportion estimates derived from quantitative study designs (i.e., cross‐sectional or longitudinal studies) referring to a specific time frame or seasonality (e.g., influenza vaccination campaigns of the previous year) were considered eligible for inclusion in the meta‐analysis. The *I* ^2^ metric was used to test heterogeneity (Higgins et al. 2003). All calculations were performed using Stata, version 17.0.

### 2.6 Quality Appraisal

The included studies were assessed for methodological validity using the standard JBI critical appraisal instruments (Munn et al. 2019) by two independent reviewers.

## 3 Results

The initial search identified 3452 records. After removing 2517 duplicates, 935 records underwent title and abstract screening. A total of 155 remaining records underwent full‐text screening. Finally, 42 remaining records were included in this review (Figure 1). Our primary goal was to provide a comprehensive synthesis of the phenomenon, ensuring a nuanced understanding of the motivators and barriers to vaccination, regardless of the study design from which individual findings emerged. To achieve this, results are presented as determinants and motivations for both vaccination hesitancy and uptake in nurses, depending on the elements explored by the included studies, as we believed this approach would offer an integrated and holistic perspective of the phenomenon. To be coherent with the existing literature on vaccination hesitancy, we referred to the following terminology along the results: confidence in vaccine safety, confidence in vaccine effectiveness, complacency, accessibility, mode of administration, experience with past vaccination, knowledge/awareness, risk/benefit.

### 3.1 Quality Appraisal

Two independent reviewers assessed the overall quality of the included studies by exploiting the JBI assessment tools and estimated it as rather high (between 70% and 100%)—only one study was scored as 62.5%.

### 3.2 Participants and Characteristics of the Included Studies

The total population size of the included studies was 28,683 participants. Some studies included students and healthcare professionals other than nurses; nurses were 22,422 in total. Among the 42 included studies (Table 2), nine were conducted in China, seven in the USA, five in the United Kingdom, three in Italy, two in France, two in Israel, two in Switzerland, one in Croatia, one in Germany, one in Australia, one in Saudi Arabia, one in Singapore, one in Slovenia, one in Nigeria, one in Greece, one in Poland, one in Cyprus, one in Jordan, and one in Ghana. Thirty‐five were analytical cross‐sectional studies, four were qualitative descriptive studies, one was a quasi‐experimental study, and two were longitudinal studies. Studies included influenza vaccination (*n* = 39, 93%), hepatitis B vaccination (*n* = 3, 7%), or a mix of multiple vaccinations together (*n* = 4, 9.5%). Six studies investigated the determinants of vaccination hesitancy, 33 studies explored the motivations for vaccination hesitancy, 13 studies investigated the determinants of vaccination uptake, and 23 studies explored the motivations for getting vaccinated (some studies investigated both determinants and motivations).

**TABLE 2:** Extracted data from included studies.

Study and design	Sample	Methods for data collection	Vaccine	Place, setting	Vaccine uptake	Determinants of vaccine hesitancy	Motivations for vaccine hesitancy	Determinants of vaccine uptake	Motivations for vaccine uptake
Alsaleem (2013) Analytical cross‐sectional study	*N* = 232 nurses	Questionnaire	H1N1	Saudi Arabia, primary healthcare centres	Overall 28%	—	*Categories emerged:* – Confidence in vaccine safety – Risk/benefit *Specifically*: – Fear of side effects (80.8%) – Uncertainty on the safety of the vaccine (70.1%) – Patients are not of high risk (37.7%)	—	– Personal protection (38.5%) – Fear of transmitting disease to family and relatives – Fear of transmitting disease to patients (36.9%)
Canning et al. (2005) Analytical cross‐sectional study	*N* = 144 nurses and healthcare assistants	Questionnaire	Influenza	United Kingdom, hospitals	7.60%	—	*Categories emerged*: – Complacency – Knowledge/awareness – Accessibility – Risk/benefit *Specifically*: – Do not think it is needed (29%) – Not aware of vaccine (18%) – ‘Did not want it’ (14%) – No time (5.5%) – Never considered it (3%) – Not available at convenient times (3%) – Did not think it was beneficial (2%)	—	– Decrease sick leave (44%) – Personal protection (28%) – Preventing the spread of the virus (15%) – Protecting patients from flu (10.5%)
Chan et al. (2021) Analytical cross‐sectional study	*N* = 1306 nurses and 23 nursing student	Questionnaire	Influenza	China (Hong Kong)	36% in the 2014/15, 35% in 2015/16, 39% in 2016/17, 43% in 2017/18, 47% in 2018/19. 39.3% non‐adherers: never vaccinated in the considered period; 39.8% partial adherers: vaccinated sometimes in the considered period; 20.9% full adherers: always vaccinated in the considered period	—	—	Full adherence was st associated with: – Female gender (adjusted odds ratio 0.60) – Age > 40 years (aOR 2.92) – Long‐term care facility nurse (aOR 0.56) – Uptake during studentship (aOR 3.83) – Local prevalence of seasonal influenza (aOR 0.51) – Expert opinion (aOR 4.04)	
Clark et al. (2009) Analytical cross‐sectional study	*N* = 1017 nurses	Questionnaire	Influenza	USA	59%	—	*Categories emerged*: – Confidence in vaccine safety – Confidence in vaccine effectiveness – Risk/benefit *Specifically*: – Concern about adverse reactions (39%) – Small chance of contracting influenza (19%) – Limited contact with high‐risk patients (18%) – Flu vaccine is not effective enough (18%) – Too busy/forgot (17%)	—	– Protect myself from illness (95%) – Protect my patients from illness (74%) – Member of target group for vaccination (19%) – Local epidemic/bad influenza season (9%) – Workplace mandate (3%)
Durovic et al. (2020) Analytical cross‐sectional study	*N* = 409 nurses	Questionnaire	Influenza	Switzerland, hospital	56%	—	*Categories emerged*: – Confidence in vaccine safety – Confidence in vaccine effectiveness – Knowledge/awareness *Specifically*: – Fear of short‐term adverse reactions (51%) – Fear of long‐term sequela (32%) – Doubt regarding efficacy (25%) – Insufficient evidence (46.9%) – Right of self‐determination (51%) – Insufficient information about vaccine (34.9%)	—	—
Festini et al. (2007) Longitudinal study	*N* = 327 nurses	Questionnaire	Influenza	Italy, Children's hospital	30.3%	—	*Categories emerged*: – Experience with past vaccination – Confidence in vaccine safety – Complacency – Risk/benefit *Specifically*: – To avoid medications when not necessary (62.4%) – The doctor suggested not to get vaccinated (10.4%) – Vaccination did not work on them in the past (8.8%) – Vaccination is risky (8.8%) – They never or rarely got the flu (8.2%) – To stay home if ill (4.9%)	—	– Not to be contagious to the patients (63%) – Not to get ill (55.6%) – To safeguard their relatives (50%) – It is convenient to get vaccinated at the workplace (46%) – Not to stay home from work (35%) – For personal health‐related condition (30%) – Yearly get vaccinated (21%) – Vaccination was free (21%) – Convinced by the vaccine campaign (13%) – Colleagues got the vaccine (2%) – Pressured to get vaccinated (1%)
Gafner et al. (2021) Analytical cross‐sectional study	(*N* = 339 healthcare professionals ( 41.4% physicians and 58.6% nurses)	Questionnaire	Influenza	Israel, hospital	69.2% of nurses were vaccinated	Factors that diminished vaccination rate for all HCPs: – Longer professional experience (*p* = 0.002) – Older age (OR 0.924, 95% CI 0.868–0.984, *p* = 0.014) – Portal or e‐mail messages (OR 0.259, 95% CI 0.098–0.687, *p* = 0.007) – Higher number of children (OR 0.337, 95% CI 0.164–0.692, *p* = 0.003)	—	– Conversations with supervising nurse (*p* < 0.001) – Conversation with the head of the ward (*p* = 0.024) – Being senior resident with a subspecialty in infectious diseases (*p* < 0.001) The 2nd and 3rd points only in medium‐size hospital.	– 8.3% pleasing my boss – 56.4% protecting my family – 49.6% to protect patients – 77.4% staying healthy
Galanis et al. (2023) Analytical cross‐sectional study	*N* = 861 nurses	Questionnaire	Influenza	Greece	57.3% of the nurses willing to accept the vaccine	– Fear of side effects because of COVID‐19 vaccination (adjusted beta: −0.22, 95% CI: −0.32 to −0.11, *p* < 0.001) – Higher levels of exhaustion due to measures against COVID‐19 (adjusted beta: −0.74, 95% CI: −0.99 to −0.49, *p* < 0.001)	—	– Older age (adjusted beta: 0.08, 95% CI: 0.01–0.15) – Higher levels of perceived support from significant others (adjusted beta: 0.39, 95% CI: 0.09–0.68)	
Henriksen Hellyer et al. (2011) Analytical cross‐sectional study	*N* = 587 nurses	Questionnaire	Influenza H1N1	USA	62%	—	*Categories emerged*: – Complacency – Confidence in vaccine safety – Confidence in vaccine effectiveness – Risk/benefit – Accessibility *Specifically*: – I don't want to be vaccinated (21%) – Worry about side effects (17%) – Unconcerned about the threat of H1N1 at the moment (11%) – Have contraindications to vaccination (5%) – Universal infection control practices are sufficient (4%) – Not the right time, I will be vaccinated later (2%) – No onsite vaccination service at the workplace (3%) – Worry that the vaccine might cause flu (1%) – The vaccine will not work (0.4%) – Dislike of the brand of vaccine offered (0.4%)		– Worry about catching swine flu/H1N1 infection (25%) – Worry about transmission of swine flu H1N1 to others (20%) – Follow the advice from health authorities (23%) – Desire to fulfil my professional obligation (16%) – Vaccination is a mandatory requirement in my workplace (20%) – Other reason (6%)
Hu et al. (2011) Analytical cross‐sectional study	*N* = 278 nurses	Questionnaire	Influenza H1N1	China, children's hospital	73.7%	—	*Categories emerged:* – Confidence in vaccine safety – Confidence in vaccine effectiveness – Complacency *Specifically*: – Worry about the safety and quality of the H1N1 vaccine (32, 42.8%) – Not concerned to be infected (12, 16.4%) – Thinking that vaccines will not work (10, 13.7%) – Worry about side effects (6, 8.2%)	—	– Wanting to prevent infection and be better protected from influenza (78.5%) – Belief that healthcare workers are the priority group of vaccination (11.7%) – Worry about contracting infection (2.9%)
Jędrzejek and Mastalerz‐Migas (2022) Analytical cross‐sectional study	*N* = 165 healthcare workers (26.1% nurses)	Questionnaire	Influenza	Poland, hospital and primary healthcare setting	Nurses in 2018/19: 35%; in 2019/20: 42%	—	*Categories emerged*: – Accessibility – Confidence in vaccine safety – Mode of administration *Specifically*: – Lack of time (25%) – Fear of vaccine adverse effects (18%) – Contraindications (10.7%) – Fear of injection (10.7%)	—	– Self‐protections (94.4%) – Protection of family/friends (61.1%) – Protection of patients (50%)
Johansen et al. (2012) Analytical cross‐sectional study	*N* = 193 nurses	Survey	Influenza	USA, hospitals, clinic, long‐term facilities	35.5% got the vaccine 10 times in the past 10 years	—	*Categories emerged*: – Confidence in vaccine safety – Complacency – Accessibility *Specifically*: – Contraindications and allergies (31.6%) – Side effects and past vaccine reactions (28.3%) – Personal choice (11.0%) – The vaccine is not necessary due to self‐immunity (6.5%) – Inconvenience (6.5%)	—	– To protect oneself (46.5%) – To protect patients (33.5%) – To decrease and prevent the incidence of influenza in general (31.6%) Additionally: – 32.3% strongly agreed that ‘flu vaccine is effective in preventing influenza in healthcare workers’ – 40.6% strongly agreed that the influenza vaccine prevents the spread of influenza to patients – 33.5% strongly agreed that the influenza vaccine is effective in preventing influenza‐related hospitalisations in vaccinated persons
Kwok et al. (2021) Analytical cross‐sectional study	*N* = 1205 nurses	Questionnaire	Influenza (and COVID‐19, which we will not consider)	China (Hong Kong)	2019–2020: 49%	—	—	Higher influenza vaccination uptake was associated with: – Older age – Presence of chronic diseases – Working in public hospitals – Having stronger vaccine confidence – Stronger collective responsibility – Weaker vaccine complacency (i.e., perceived the disease as low risk) – Weaker constraints (i.e., perceived low vaccine availability, affordability, and accessibility) – Weaker calculation (i.e., engagement in information searching)	
Lau et al. (2020) Analytical cross‐sectional study	*N* = 753 nurses	Online questionnaire	Influenza	China (Hong Kong)	44% in 2017/18	– Not received influenza vaccination in the previous season – Never been vaccinated in the past 5 years (88% of participants) – No intention to be vaccinated in the future, being sceptical and critical towards safety and effectiveness of influenza vaccine (98%)		– High seasonal influenza vaccination uptake – Very strong future vaccination intention (81%) – Great confidence in the effectiveness of influenza vaccine (77%)	
Lecce et al. (2022) Quasi‐experimental study	*N* = 4165 healthcare workers who got vaccinated during the campaign (exact number of nurses not available)	Questionnaire	Influenza	Italy, hospital	67.5% (*n* = 2381) received influenza vaccine, among which 414 were nurses (17.4% of the HCWs who received the influenza vaccine)	—	—	—	– Vaccination is the most effective strategy of prevention (78%) – As a healthcare professional it is my duty to get vaccinated to protect patients (37.4%) – As a healthcare worker I am more exposed to the flu (14.5%)
Leung et al. (2022) Analytical cross‐sectional study	*N* = 1193 nurses	Online survey	Influenza (and COVID‐19, which we will not consider)	China (Hong Kong)	Uptake 2020: 49.5% Intention 2021: mean 2.78 (±1.26) (1 = definitely not; 5 = definitely yes)	—	—	On a 7‐point scale, nurses had: – Relatively high scores in confidence (*M* = 4.93, SD = 1.20), calculation (*M* = 5.62, SD = 0.86) and collective responsibility (*M* = 5.28, SD = 1.14) – Low scores in complacency (*M* = 3.63, SD = 1.21) and constraints (*M* = 3.13, SD = 1.25). Additionally: – All the 5C factors were intercorrelated and related with vaccination intention – Calculation was positively correlated with vaccination intention (*p* < 0.001)	
Lewthwaite et al. (2014) Analytical cross‐sectional study	*N* = 310 nurses	Questionnaire	Influenza; H1N1	United Kingdom, hospitals	37% both; H1N1 only 16%; influenza only 13%	—	Influenza: *Categories emerged*: – Accessibility – Knowledge/awareness – Risk/benefit – Confidence in vaccine safety – Experience with past vaccination *Specifically*: – Minor illness (15%) – Not at risk (23%) – Not offered (11%) – Unwell after previous vaccine (15%) – Fear of side effects (24%) H1N1: *Categories emerged:* – Risk/benefit – Confidence in vaccine safety – Accessibility *Specifically*: – Minor illness (11%) – Not at risk (11%) – Not offered (11%) – Unwell after previous vaccine (3%) – Fear of side effects (33%) – Safety (19%)	—	– Fear for the health of family members – Previous bad experience of influenza
Livni et al. (2008) Analytical cross‐sectional study	*N* = 217 nurses	Questionnaire	Influenza	Israel, paediatric hospital	35.20%	—	*Categories emerged*: – Confidence in vaccine effectiveness – Confidence in vaccine safety – Mode of administration *Specifically*: – Doubt the vaccine's effectiveness (61.2%) – Forgot/missed the deadline (8.3%) – Afraid of vaccine‐associated influenza (8.3%) – Disturbed by potential side effects (5.3%) – Against vaccination in principle (4.5%) – Dislike the needles (0.8%) – Other or more than one reason (9.1%)	Uptake of the influenza vaccine was associated with: – Good medical knowledge – Longer duration of employment	– To protect myself (65%) – To comply with recommendations (25%) – To protect my family (5%) – To serve as a model (2%) – More than one reason (13%)
Luo et al. (2021) Analytical cross‐sectional study	*N* = 1412 healthcare workers (741 nurses)	Online and face‐to‐face survey	Influenza	China, hospital	In nurses = 18.35%	—	*Categories emerged*: – Accessibility – Complacency – Confidence in vaccine safety – Experience with past vaccination *Specifically*: – No time (67.60%) – It is unnecessary to get vaccinated (26.78%) – Worry about the quality of influenza vaccine (26.61%) – Worry about the adverse reactions after vaccination (28.10%) – The price of influenza vaccine is too high (18.02%)	– Nurses working in sentinel hospitals had a higher vaccination rate than those in non‐sentinel hospitals – Nurses working in high‐risk departments had a higher vaccination rate than those working in other departments (22.92% vs. 13.08%) – Nurses with a positive attitude had a higher vaccination rate than those who had a negative attitude (23.84 vs. 9.72) – Nurses with good knowledge had a higher vaccination rate than those with poor knowledge (22.78% vs. 15.06%)	– Protecting oneself from influenza (94.85%) – Recommendation from government and health authorities (44.85%) – Recommendation by relatives or friends (38.97)
McEwen and Farren (2005) Analytical cross‐sectional study	*N* = 246 nurses	Questionnaire	HBV and influenza	USA	HBV 92%, influenza 86% had ever received a flu shot, while 69% received it in the 2 of the previous 4 years	HBV: not vaccinated nurses were older than the vaccinated group (mean age for getting the vaccine = 46.3 vs. 52.5 for not getting the vaccine, *p* = 0.01)	For HBV vaccine: *Categories emerged*: – Confidence in vaccine safety – Confidence in vaccine effectiveness – Complacency – Accessibility *Specifically*: – Not working in nursing (42.1%) – Not working in an area considered high risk for exposure (31.6%) – Concern on side effects (31.6%) – Vaccine expense (15.8%) – Concern that vaccine not effective (5%) – Personal religious beliefs (5%) – Previous hepatitis B infections (5%) For influenza vaccine: *Categories emerged*: – Confidence on vaccine safety – Complacency – Experience with past vaccination *Specifically*: – Concern about side effects (37.3%) – Not concerned about getting the flu (29.9%) – Ill in the past despite receiving a flu shot (28.4%)	All nurses licensed for ≤ 15 years received the HBV	– Belief that it is effective in preventing the flu (81.8%) – It is provided by employer free of charge (75.1%) – Concern about being at risk for exposure (66.3%) – Work with clients who are high risk (44.2%) – Had the flu in the past and do not want to experience it again (37.6%) – Being over 50 years of age (35.4%)
Ofstead et al. (2008) Analytical cross‐sectional study	*N* = 513 nurses	Questionnaire	Influenza	USA, tertiary medical centre	Most respondents (86.7%) previously received influenza vaccine, 64.5% intended to receive vaccine in the next season	—	– Vaccine should be used for people at higher risk (62.7%) – Fear of vaccination side effects (57.1%) – Not at high risk for influenza (44.4%) – Dislike receiving injections (39.4%) – Influenza vaccine is not effective (31.3%) – Did not have time to get vaccinated (8.1%)	– Not believing that the injected influenza vaccine contained live viruses	
Omotowo et al. (2018) Analytical cross‐sectional study	*N* = 580 nurses	Questionnaire	HBV	Nigeria, hospital	—	—	*Categories emerged*: – Accessibility – Knowledge/awareness – Complacency *Specifically*: – Cost (5.8%) – Don't know where to take the vaccine (28.9%) – Don't believe I could be infected (2.5%) – Others (e.g., long vaccination schedule and lack of time) (15.7%)	—	—
O'Reilly et al. (2005) Analytical cross‐sectional study	*N* = 203 nurses	Questionnaire	Influenza	United Kingdom, elderly care	37%	—	*Categories emerged*: – Complacency – Confidence in vaccine safety *Specifically*: – No personal benefit as being healthy (69%) – Concern about side effects (19%)	—	– Protect oneself against influenza (96%) – Protect patients (14%)
Papageorgiou et al. (2022) Analytical cross‐sectional	*N* = 962 healthcare workers (538 nurses)	Questionnaire	Influenza	Cyprus, hospital	Nurses uptake flu vaccine (2019–20): 106 (20%)	—	*Categories emerged*: – Confidence in vaccine safety – Confidence in vaccine effectiveness – Knowledge/awareness – Experience with past vaccination *Specifically*: – No particular reason – Afraid of complications – Not believe that flu vaccine helps – Last time I had the vaccine I got sick – Flu is not a serious illness and there is no need to get vaccinated – Flu vaccine is not working – Flu vaccine can cause the disease – Not enough information about the flu vaccine – Against vaccination in general – Not at risk of getting the flu – No time to go for vaccination	—	– To protect family – To protect myself from flu complications – Being at greater risk of getting the flu being an HCW – Being a HCW, I pose greater risk for transmitting the disease to fragile populations – It is HCWs' duty to protect patients from flu – Flu can cause serious disease and complications – Motivated by the fact that colleagues had the vaccine
Pavlič et al. (2020) Qualitative study	*N* = 19 nurses	Semi‐structured interviews	Influenza	Slovenia	0%	—	*Categories emerged*: – Confidence in vaccine effectiveness – Confidence in vaccine safety – Complacency – Risk/benefit *Specifically:* – Doubt regarding the vaccine effectiveness – Potential side effects – Belief that young HCWs are well protected and not at high risk – Overrated trust in their immune systems – Believe pharmaceutical industry marketing was targeting them	—	—
Pinto et al. (2019) Analytical cross‐sectional study	*N* = 140 nurses	Questionnaire	Influenza + vaccination in general	Italy, Children's hospital	19.3% for influenza	—	Influenza *Categories emerged*: – Risk/benefit – Confidence in vaccine safety – Knowledge/awareness *Specifically*: – Belief that my risk of contracting influenza is low (27%) – Belief that the risk of transmitting influenza to patients is low (22.1%) – Afraid of damages caused by the influenza vaccination (13%) – Limited information on the vaccine (36%) Vaccine in general: *Categories emerged*: – Risk/benefit – Confidence in vaccine safety – Confidence in vaccine effectiveness *Specifically*: – Belief that my risk of contracting vaccine‐preventable disease is low (38%) – Belief that the risk of transmitting a vaccine‐preventable disease to a patient is low (31%) – Vaccines' benefits are uncertain (22%) – Afraid of vaccine side effects (22.8%)	—	—
Pless et al. (2017) Qualitative study	*N* = 18 nurses	Semi‐structured interviews	Influenza	Switzerland	0%	—	*Specific categories emerged*: – Maintaining a strong and healthy body – Protecting decisional autonomy – To take care of themselves and others – Perception of an untrustworthy environment	—	—
Rhudy et al. (2010) Qualitative descriptive study	*N* = 14 nurses	Semi‐structured interviews	Influenza	USA, hospital	0%	—	*Categories emerged*: – Confidence in vaccine effectiveness – Confidence in vaccine safety – Accessibility – Complacency *Specifically*: – Low priority – Sense of good health – Scepticism of the vaccine's value – Fear of vaccine side effects – Hand washing as prevention – Inconvenient immunisation locations	—	—
Sallam et al. (2022) Analytical cross‐sectional study	*N* = 1218 healthcare workers (412 nurses)	Self‐administered electronic survey	Influenza	Jordan	Nurses uptake flu vaccine (2020–21): 70.9%	– Have to pay for the vaccine (51.7%) (*p* < 0.001)	—	—	– Vaccine provided for free (64.6% of nurses) – 83.5% of nurses: vaccinating HCWs against flu helps protect patients from severe illness/death – 83.3% Influenza vaccination reduces absenteeism from work
Seale et al. (2011) Analytical cross‐sectional study	*N* = 1044 nurses	Questionnaire	H1N1	China, hospitals	22%	—	*Categories emerged*: – Confidence in vaccine safety *Specifically*: – Concerns on the side effects of vaccine (64%) – The vaccine may have not been tested adequately (47%) – Vaccine may cause the flu (23%)	—	– The vaccine will protect from the flu (45%) – The flu vaccine will stop the spread of flu (52%)
Smedley et al. (2007) Analytical cross‐sectional study	*N* = 1890 nurses	Questionnaire	Influenza	United Kingdom, hospitals		—	*Categories emerged*: – Confidence in vaccine safety – Accessibility – Confidence in vaccine effectiveness – Knowledge/awareness *Specifically*: – Concern about side effects (38%) – Lack of time (16%) – Doubts about efficacy for protecting self or others (29%) – Unaware of availability or access to vaccine (10%)	—	– Protected time or easier access (37%) – Financial rewards (14%) – Having received more information about side effects or efficacy (38%) – Having received more information on effect on winter workload or staff absence (29%)
Smith et al. (2016) Analytical cross‐sectional study	*N* = 85 nurses	Online survey	Influenza, H1N1	Australia, General Practices	78.8% influenza; 63.5% H1N1	—	*Categories emerged*: – Confidence in vaccine safety – Complacency – Accessibility – Mode of administration *Specifically*: – Concerns about allergies or contraindications (45.9%) – Not needing the vaccine/having natural immunity (28.3%) – Concern about side effects (23.5%) – No time or difficult access (14.1%) – Dislike of injections (10.6%) – Vaccination gives influenza (2.4%)	—	– Self‐protection (60.0%) – Protection of others (2.7%) – Protection of family (22.4%) – Having a medical condition (21.2%) – Working in high‐risk areas (21.2%) – To decrease the spread of influenza (21.2%) – Protection of consumers (18.8%)
Tagbor et al. (2023) Analytical cross‐sectional study	*N* = 160 nurses	Questionnaire	HBV	Ghana, hospital	98.80% started the HBV process, 99.40% continued it, 100% completed it.	– Perceived susceptibility (*r* = 20.13, *p* = 0.05) – Perceived barriers (*r* = 20.24, *p* = 0.01) (i.e., vaccination ineffectiveness, time constraints, high costs and side effects)	—	– Nurse perceived benefit (*r* = 0.14, *p* = 0.05) – Cues to action (*r* = 0.17, *p* = 0.05)	—
To et al. (2010) Analytical cross‐sectional	*N* = 812 nurses	Questionnaire	Influenza; H1N1	China (Hong Kong)	Influenza: 37.5% considered it, 40.8% rejected it, 21.6% undecided. H1N1: 13.3% considered it, 45.4% rejected it, 41.3% undecided	—	*Categories emerged*: – Confidence in vaccine safety – Confidence in vaccine effectiveness – Complacency *Specifically*: – Concerns about side effects of the vaccine (65%–68%) – Perceived mild nature of influenza (26.3%–30.8%) – Belief that the vaccine could not prevent infection (55.9%–58.5%)	—	– Vaccination could protect against infection (85%) – Work requirements and protecting others in the workplace (50%)
Toh et al. (2012) Analytical cross‐sectional study	*N* = 183 nurses	Questionnaire	H1N1	Singapore, Primary care clinics	39.90%	—	*Categories emerged*: – Confidence in vaccine safety – Confidence in vaccine effectiveness – Mode of administration – Complacency *Specifically*: – Fear of side effects (51.4%) – Unsure of effectiveness (59.7%) – Dislike getting injections (19.4%) – Feel not at high risk of getting complications from H1N1 (11.1%) – Medical reasons (12.5%) – Believe to be immune to H1N1 (5.6%)	—	—
Tomljenovic et al. (2021) Analytical cross‐sectional study	*N* = 324 healthcare workers (181 nurses)	Questionnaire	Measles, flu, HPV	Croatia	Flu vaccine: 53.4% never received it, 30.3% repeatedly received it, 16.3% receive it yearly. Measles vaccine: 62.4% yes, 20.5% don't know, 17.1% no	—	*Categories emerged*: – Complacency – Confidence in vaccine safety *Specifically*: – Already protected by constant exposure (38%) – No medical indication (20%) – Other (14%) – Side effects (12%) – Not effective (11%) – Forgot (5%)	—	—
Wicker et al. (2009) Longitudinal study	*N* = 1040 nurses	Questionnaires	Influenza	Germany, hospital	17.40%	—	*Categories emerged*: – Complacency – Confidence in vaccine effectiveness – Confidence in vaccine safety – Mode of administration *Specifically*: – Influenza is not a serious illness (18.4%) – Vaccine does not provide sufficient protection (26.9%) – Fear of adverse reaction (6%) – Fear of injection (6.6%) – Vaccine causes influenza (21.7%)	—	—
Willis and Wortley (2007) Qualitative study	*N* = 71 nurses	8 focus group. Focus Group Discussion Guide was developed specifically for this study by CDC in collaboration with the Academy for Educational Development	Influenza	USA	—	—	*Categories emerged*: – Confidence in vaccine safety – Confidence in vaccine effectiveness – Complacency – Knowledge/awareness. *Specifically*: – Concerns regarding the safety of the vaccine – Concerning regarding the lack of information on vaccine effectiveness – Belief of not being at risk for influenza because of a stronger immune system because of workplace exposure – Belief that the vaccine was not important and that using routine preventive measures minimises the risk of contracting influenza	—	—
Wilson et al. (2019) Analytical cross‐sectional study	*N* = 1539 nurses	Questionnaire	Influenza	France, Hospital and communities	28%	—	*Categories emerged*: – Experience with past vaccination – Complacency – Confidence in vaccine safety *Specifically*: – Ill despite influenza vaccination (19.8%) – Forgot to get vaccinated, negligence, lack of time (7.1%) – Does not believe they are susceptible to influenza (42.9%) – Preference towards homeopathy (36.7%) – Contraindications to the vaccine (2.9%) – Fear of adverse effects (40.6%)	—	– To protect patients – To protect relatives – To contribute to the herd immunity – To protect myself
Wilson et al. (2020) Analytical cross‐sectional study	*N* = 1539 nurses	Questionnaire	All vaccinations	France, Hospital and communities	96% received BCG vaccine, 73% the dTPolio booster vaccine, 61% ≥ 3 HBV doses, 58% pertussis, 64% measles, 39% varicella, 27% influenza. Community nurses < vaccination uptake (except for varicella and influenza)	—	– A causal link between the HBV and multiple sclerosis was considered likely or very likely by 57% of nurses – 34% for a link between aluminium adjuvants and Alzheimer's disease, 14% for a link between measles vaccine and autism	—	—
Wong et al. (2010) Analytical cross‐sectional study	*N* = 267 nurses	Questionnaire	H1N1	China (Hong Kong), community	27%	—	*Categories emerged*: – Confidence in vaccine safety – Confidence in vaccine effectiveness *Specifically*: – Concerns on effectiveness (59%) – Side effects (83%) – Production site (27%)	– Having been vaccinated for influenza in the previous 12 months was significantly associated with the willingness to accept influenza A (H1N1) vaccination (OR = 4.03, 95% CI: 2.03–7.98)	—
Zhang et al. (2011) Analytical cross‐sectional study	*N* = 522 nurses	Questionnaire	Influenza, H1N1	United Kingdom	36%	—	*Categories emerged*: – Confidence in vaccine safety – Confidence in vaccine effectiveness – Complacency – Accessibility – Mode of administration *Specifically*: – Concern about side effects: 63% – No need (46%) – Concerns about vaccine's effectiveness/safety (35.2%) – No time or difficult to access vaccination (19%) – Dislike of injection or fear of pain (14%) – Personal choice (2%) – Negative reports from media/press (2%)	– Several risk perception items including personal vulnerability to influenza or H1N1, mortality risk of H1N1, and the likelihood of transmitting influenza to patients were predictors of vaccination uptake. Nurses with a high knowledge level were more likely to get vaccinated compared to those with a low knowledge level	– To protect oneself (57%) – To protect patients (26%) – To protect family/friends/children (16.2%) – Health requirement (14%) – To avoid sick leave (14%) – Working in high‐risk areas (12%)

### 3.3 Vaccination Hesitancy

#### 3.3.1 Determinants of Vaccination Hesitancy

Six studies assessed the factors associated with vaccination hesitancy. Among them, the most commonly reported factors associated with vaccine hesitancy were: confidence in vaccine safety (Galanis et al. 2023; Lau et al. 2020; Tagbor et al. 2023), confidence in vaccine effectiveness (Lau et al. 2020; Tagbor et al. 2023), older age (Gafner et al. 2021; McEwen and Farren 2005), longer professional experience (Gafner et al. 2021; McEwen and Farren 2005), and cost associated with the vaccine (Sallam et al. 2022; Tagbor et al. 2023). Other less common determinants of vaccination hesitancy identified by some authors were portal/e‐mail invitations (Gafner et al. 2021), higher number of children (Gafner et al. 2021), time constraints (Tagbor et al. 2023), and not having received the vaccination in the past (Lau et al. 2020).

#### 3.3.2 Motivations for Vaccination Hesitancy

**FIGURE 2:**
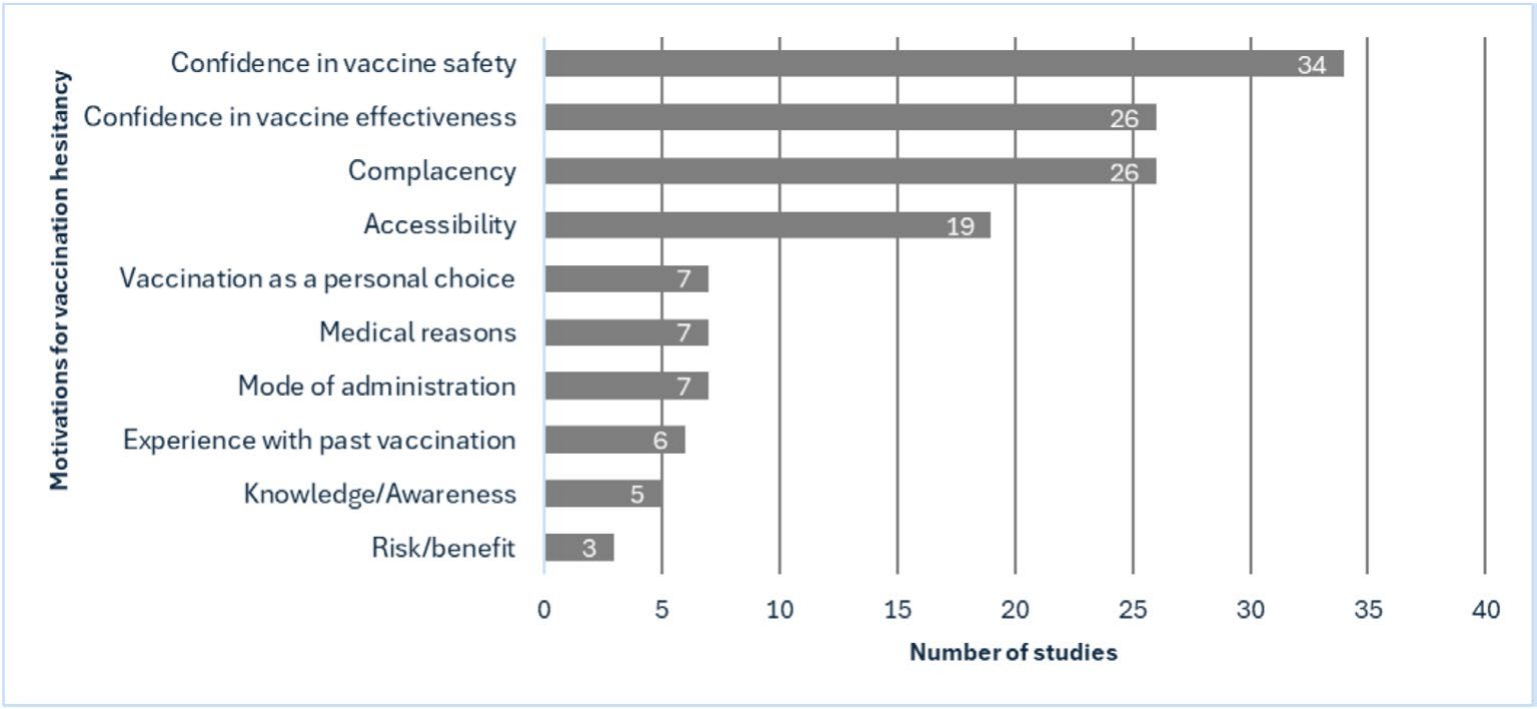
Motivations for vaccination hesitancy reported by the included studies. [Colour figure can be viewed at wileyonlinelibrary.com]

Thirty‐three studies (Alsaleem 2013; Canning et al. 2005; Clark et al. 2009; Durovic et al. 2020; Festini et al. 2007; Henriksen Hellyer et al. 2011; Hu et al. 2011; Jędrzejek and Mastalerz‐Migas 2022; Johansen et al. 2012; Lewthwaite et al. 2014; Livni et al. 2008; Luo et al. 2021; McEwen and Farren 2005; Ofstead et al. 2008; Omotowo et al. 2018; O'Reilly et al. 2005; Papageorgiou et al. 2022; Pavlič et al. 2020; Pinto et al. 2019; Pless et al. 2017; Rhudy et al. 2010; Seale et al. 2011; Smedley et al. 2007; Smith et al. 2016; To et al. 2010; Toh et al. 2012; Tomljenovic et al. 2021; Wicker et al. 2009; Willis and Wortley 2007; Wilson et al. 2019, 2020; Wong et al. 2010; Zhang et al. 2011) explored the motivations for vaccination hesitancy (Figure 2). In all the included studies, nurses reported being hesitant towards vaccination because of their confidence in vaccine safety, mainly related to the fear of side effects and adverse reactions of the vaccine. In most studies (*n* = 26, 76%) nurses reported being hesitant towards vaccination because of their confidence in vaccine effectiveness and because of complacency reasons (they considered they did not need to get vaccinated or they did not fear contracting the virus). In around half of the studies (*n* = 19, 55%), nurses reported they did not receive/plan to receive the vaccine because of accessibility reasons (which mainly included time constraints, inconvenient location, or high costs for the vaccine). Other reasons reported by the nurses for being vaccine hesitant were considering vaccination as a personal choice or being against it in principle; medical reasons (e.g., contraindications); mode of administration (i.e., dislike or fear of injections); experience with past vaccination (e.g., ill despite/after vaccination); having not received enough information on the vaccine; and the perceived risk/benefit (e.g., belief that they had a low risk of transmitting the infection to other people if they contracted it). Motivations for vaccination hesitancy are summarised in Figure 2.

### 3.4 Vaccination Uptake

#### 3.4.1 Determinants of Vaccination Uptake

**FIGURE 3:**
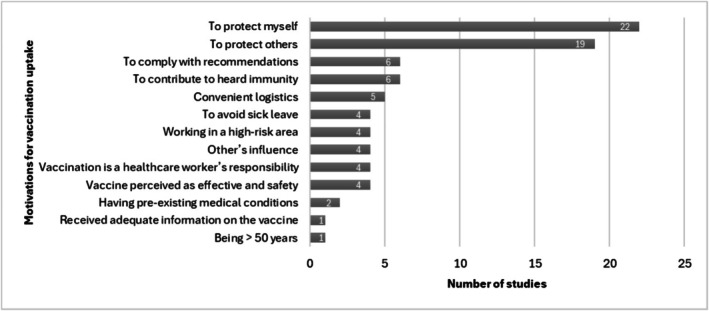
Motivations for vaccination uptake reported by the included studies.

Thirteen studies assessed the factors associated with vaccination uptake (Figure 3). Among them, the most common reported factors positively associated with vaccine uptake were having strong confidence in the vaccine benefits (Kwok et al. 2021; Lau et al. 2020; Leung et al. 2022; Tagbor et al. 2023), having a high sense of collective responsibility (Kwok et al. 2021; Leung et al. 2022; Tagbor et al. 2023), previous vaccination uptake/positive intentions towards future vaccination (Chan et al. 2021; Lau et al. 2020; Wong et al. 2010), having weaker vaccine complacency (i.e., not perceiving the disease as low risk) (Kwok et al. 2021; Leung et al. 2022; Zhang et al. 2011), having less constraints towards vaccination (i.e., not experiencing issues related to vaccine availability, affordability or accessibility) (Kwok et al. 2021; Leung et al. 2022), having good medical knowledge (Livni et al. 2008; Luo et al. 2021; Zhang et al. 2011), older age (Chan et al. 2021; Galanis et al. 2023; Kwok et al. 2021), and valuing experts' opinion (Chan et al. 2021; Gafner et al. 2021). Other less common factors positively associated with vaccination uptake identified by some authors included higher local prevalence of the virus (Chan et al. 2021), female gender (Chan et al. 2021), having a chronic disease (Kwok et al. 2021), and having a positive attitude (Luo et al. 2021). Some authors also reported that the area of employment was associated with vaccination uptake, with nurses working either in higher risk departments (Luo et al. 2021), infective diseases areas (Gafner et al. 2021), long‐term facilities (Chan et al. 2021), or public hospitals (Kwok et al. 2021) reporting higher vaccination uptake. Two studies reported that calculation (i.e., engagement in information searching) was associated with vaccination uptake, although one reported a positive association (Leung et al. 2022) and one reported a negative association (Kwok et al. 2021). Two studies reported that duration of employment was associated with vaccine uptake. However, one of them reported that longer duration of employment was associated with vaccination uptake (Livni et al. 2008) while the other (McEwen and Farren 2005) reported the opposite. Galanis et al. (2023) also found that higher levels of perceived support from significant others were associated with greater vaccination uptake in nurses. Finally, in one study, authors found that not believing that the vaccine contained live virus was positively associated with vaccination uptake (Ofstead et al. 2008).

#### 3.4.2 Motivations for Vaccination Uptake

Twenty‐three studies (Alsaleem 2013; Canning et al. 2005; Clark et al. 2009; Festini et al. 2007; Gafner et al. 2021; Henriksen Hellyer et al. 2011; Hu et al. 2011; Jędrzejek and Mastalerz‐Migas 2022; Johansen et al. 2012; Lecce et al. 2022; Lewthwaite et al. 2014; Livni et al. 2008; Luo et al. 2021; McEwen and Farren 2005; O'Reilly et al. 2005; Papageorgiou et al. 2022; Sallam et al. 2022; Seale et al. 2011; Smedley et al. 2007; Smith et al. 2016; To et al. 2010; Wilson et al. 2019; Zhang et al. 2011) explored the motivations for vaccination uptake. In most studies, nurses reported being willing to receive the vaccine to protect themselves (*n* = 22 studies, 96%) and/or others, mainly families and patients (*n* = 19 studies, 93%). In several studies, nurses reported they intended to receive the vaccine to contribute to herd immunity (*n* = 6 studies, 26%), to comply with recommendations (*n* = 6 studies, 26%), or because of convenient logistics (*n* = 5 studies, 22%). Some other studies reported that nurses were motivated to receive the vaccine because they perceived it as effective and safe (*n* = 4 studies, 17%), they considered vaccination as a healthcare worker's responsibility (*n* = 4 studies, 17%), of other people's influence (*n* = 4 studies, 17%), they were working in high‐risk areas (*n* = 4 studies, 17%), or to avoid sick leave (*n* = 4 studies, 17%). Few studies (*n* = 2, 9%) reported that nurses were more motivated to receive the vaccine when they had pre‐existing medical conditions. Finally, one study reported that being > 50 years old motivated some nurses to receive the vaccine; and another study reported that nurses were more motivated to receive the vaccine if they received adequate information about its efficacy, side effects, and its potential influence on winter workload and staff absence. Motivations for vaccination uptake are summarised in Figure 3.

#### 3.4.3 Proportion Meta‐Analysis of Influenza Vaccination Uptake in Nurses

Twenty‐four studies comprising a total sample of 13,370 nurses were included in the proportion meta‐analysis of influenza vaccination uptake. The pooled prevalence of influenza vaccine uptake among nurses was 44% (95% CI: 35%–73%) (Figure S1). Heterogeneity across studies was very high, with an *I* ^2^ value of 99.3%.

## 4 Discussion

This systematic review aimed to explore the determinants and motivations of vaccination hesitancy and uptake among nurses. Motivators for vaccination hesitancy and for vaccination uptake were explored by most of the studies, some studies investigated the determinants of vaccine uptake and few studies investigated the determinants of vaccine hesitancy. The most common determinants of vaccine hesitancy were older age, longer professional experience, confidence in vaccine safety and effectiveness, and costs associated with the vaccine. The most common motivations for being vaccine hesitant included confidence in vaccine safety mainly related to the fear of side effects and adverse reactions of the vaccine, confidence in vaccine effectiveness, complacency (perception of not being in need to get vaccinated, no fear of contracting the virus), and accessibility (i.e., logistics issues mainly including time constraints, inconvenient location, or high costs for the vaccine). The most common determinants of vaccine uptake were having strong confidence in the vaccine benefits, having a high sense of collective responsibility, previous vaccination uptake/positive intentions towards future vaccination, weaker vaccine complacency (i.e., not perceiving the disease as low risk), low constraints towards vaccination (i.e., not experiencing issues related to vaccine availability, affordability or accessibility), good medical knowledge, older age, and valuing experts' opinions. The most common motivations for vaccine uptake included the willingness to protect themselves and/or others, contribute to the herd immunity, and comply with recommendations. We found a pooled prevalence of influenza vaccine uptake among nurses of 44%, which is coherent with previous meta‐analyses on vaccination coverage in healthcare professionals reporting similar rates (Fan et al. 2023; Pereira et al. 2017).

These results are generally coherent with the literature and, in particular, with the matrix of determinants of vaccine hesitancy developed by the SAGE Working Group on Vaccine Hesitancy (2014), which identifies contextual, individual, group, and vaccine‐specific factors as able to influence vaccine hesitancy. Coherent with such a matrix, in our systematic review we found that, in some cases, nurses were vaccine hesitant because of knowledge/awareness reasons (i.e., insufficient information received or available on the vaccine). Some studies also reported that nurses were vaccine hesitant specifically because of their feelings of an untrustworthy environment (e.g., authorities) (Pless et al. 2017), production site (Wong et al. 2010), and that pharmaceutical industry marketing was targeting them (Pavlič et al. 2020). Individual and group factors include personal and family members' experience with vaccination, beliefs about health and prevention, medical knowledge, trust in the health system, perceived risk/benefit, perceptions about immunisation as a social norm versus not needed/harmful. In our systematic review it was markedly evident that nurses were vaccine hesitant because of their lack of confidence in vaccine safety and effectiveness, and because of their complacency (i.e., they did not perceive the need of getting protected through a vaccine). Various studies also reported that nurses tended to avoid vaccine either because they considered it to be a personal choice (Johansen et al. 2012; Zhang et al. 2011), because they wanted to protect their autonomous decision making (Pless et al. 2017) and self‐determination (Durovic et al. 2020), or because they were against vaccination in principle (Livni et al. 2008; Papageorgiou et al. 2022). Several studies also reported that nurses were vaccine hesitant because of negative experience with past vaccination (Festini et al. 2007; Johansen et al. 2012; Lewthwaite et al. 2014; McEwen and Farren 2005; Papageorgiou et al. 2022; Wilson et al. 2019), and few studies also reported that medical knowledge was associated with vaccine uptake (Livni et al. 2008; Luo et al. 2021; Zhang et al. 2011). The third factors identified by the SAGE Working Groups are vaccine‐specific issues, such as the mode of administration and delivery, the vaccination schedule, costs, and the strength of recommendation. In our systematic review several studies reported that nurses were often vaccine hesitant because of accessibility reasons such as lack of time or inconvenient location.

The results of this systematic review also align with the *Confidence, Complacency, Convenience Model of Vaccine Hesitancy* by the SAGE Working Group on Vaccine Hesitancy (2014), which describes vaccine hesitancy as resulting from the intersection between Confidence (i.e., trust in the effectiveness and safety of vaccines), Complacency (i.e., low perceived risk of vaccine‐preventable diseases and vaccination not deemed as necessary), and Convenience (i.e., extent to which availability, affordability, and willingness‐to‐pay affect vaccine uptake). In our systematic review, some studies (Kwok et al. 2021; Leung et al. 2022) specifically reported that nurses with weaker vaccine complacency had higher vaccine uptake, meaning that when nurses did not perceive the diseases as low risk, they were more prone to getting it. The same studies (Kwok et al. 2021; Leung et al. 2022) also reported that nurses with low convenience (also referred to as *constraints*) had higher vaccine uptake, meaning that when nurses did not perceive vaccines as unavailable or unaffordable, they were more prone to getting it. Although several studies did not name some variables with the above terms, they too reported determinants and motivations that fit into this 3C model. For instance, numerous studies found that nurses were vaccine hesitant because of their lack of confidence in vaccine safety and effectiveness, and that nurses who perceived vaccines as effective and safe were more prone to receive them. In several studies, nurses who considered not to be in need to get vaccinated or who did not fear contracting the virus were more hesitant towards vaccination. Also, availability (e.g., convenient locations) and affordability (e.g., reasonable cost) of the vaccine were among the aspects cited by some nurses in various studies to influence their willingness to receive the vaccine.

The studies included in this systematic review reported some factors both as motivators and determinants of vaccination hesitancy or uptake but did not specifically investigate the potential mediating or moderating role of some factors between the intention to get vaccinated and the actual uptake of the vaccine. However, the results of this systematic review are in line with the WHO's framework of behaviours and social drivers of vaccination (World Health Organization 2022b), which proposes that social processes and ‘thinking and feeling’ factors first influence the motivation to get vaccinated, and then practical issues (e.g., availability) further influence if the person will eventually receive the vaccination. As mentioned above, several studies included in this review reported that factors such as fear of vaccine side effects and vaccine effectiveness (i.e., ‘thinking and feeling’ factors), healthcare professionals and families' recommendations (i.e., social processes), availability, and affordability of the vaccine (i.e., practical issues) influenced the intention of nurses to receive the vaccine. Thus, the results of this systematic review not only contribute to strengthen the WHO's framework of vaccination behavioural and social drivers, but also underline that the elements identified by the framework applies to healthcare professionals and, specifically, to nurses.

Both the SAGE and WHO's frameworks of vaccine hesitancy were not specifically developed for nurses or healthcare professionals. However, although different determinants and motivations emerged in different extents, the results of this systematic review appear to be in line and fit into those frameworks. This shows that nurses' behaviours regarding their vaccination intentions do not excessively differ from those of the general population, even though they are healthcare professionals.

## 5 Conclusion

This systematic review represents a synthesis of the literature on vaccine hesitancy and uptake among nurses, and the results contribute to a further understanding of the beliefs, barriers and facilitators among nurses towards vaccination. Older age, longer professional experience, lack of confidence in vaccine safety, and effectiveness and accessibility (cost associated with the vaccine) were among the most common determinants of vaccine hesitancy. Lack of confidence in vaccine safety, complacency (perception of not being in need to get vaccinated), and accessibility (logistic issues) were among the most common motivations for being vaccine hesitant. Strong confidence in vaccine safety and effectiveness, a high sense of collective responsibility, previous vaccination uptake/positive intentions towards future vaccination, weaker vaccine complacency, and older age were among the most common determinants of vaccine uptake. Willingness to protect themselves and/or others, contribute to the herd immunity, and comply with recommendations were among the most common motivations for vaccine uptake in nurses.

The results of this systematic review allow a better understanding of the perceived barriers and facilitators among nurses towards vaccination. In this way, this study may represent a great support for clinicians and policy makers because it can facilitate the immediate implementation of the most‐available strategies to reduce barriers and improve facilitators. Additionally, this study may also represent a great support for researchers because it can spur future studies to identify new and effective strategies to reduce barriers and further improve facilitators to eventually increase vaccine uptake among nurses.

## 6 Strengths and Limitations

In this systematic review, the focus placed both on determinants and on motivations, as well as both of vaccine hesitancy and uptake, contributed to drawing a broad picture of the phenomenon of interest. In some cases, although very rare, some of the data were computed on healthcare professionals as a whole group and it was not possible to consider those referred to nurses only (i.e., in Gafner et al. it was not possible to explore determinants of hesitancy for nurses only, although it was for determinants and motivations for uptake) (Gafner et al. 2021). We included several studies from different countries and cultures: although vaccination is a global phenomenon, socio‐cultural and economic variables may exert different impacts on vaccination intentions.

## Author Contributions

Conceptualisation: G.L., M.L., D.F., B.A., A.C., M.C., E.R., A.M., and D.A.; Data curation: G.L.; Formal analysis: G.L. and M.L.; Investigation: G.L. and M.L.; Methodology: G.L., M.L., B.A., A.C., M.C., A.M., and D.A.; Project administration: G.L., M.L., and D.A.; Supervision: M.L. and D.A.; Visualisation: G.L.; Writing – original draft: G.L.; Writing – review and editing: G.L., M.L., D.F., B.A., A.C., M.C., A.M., and D.A.

## Conflicts of Interest

The authors declare no conflicts of interest.

## Supporting information


Appendix S1

Appendix S2

Figure S1

## Data Availability

The data that support the findings of this study are available on request from the corresponding author. The data are not publicly available due to privacy or ethical restrictions.
