# Supplementary material for: Determinants and Motivations of Vaccination Hesitancy and Uptake in Nurses: A Systematic Review and Meta‐Analysis
Source: J Clin Nurs. 2025 Jun 25;34(10):4005–37. doi: 10.1111/jocn.17852 (PMC12409242; doi:10.1111/jocn.17852)
Supplement: Supplementary file 1 — Appendix S1 [file JOCN-34-4005-s002.docx]

**Appendix S1 – Search Strategies** (searches conducted on February 2023)

| **Pubmed** | |
| --- | --- |
| **Search strategy** | **N. of records** |
| (vaccin*[Title/Abstract]) AND (Hesitancy[Title/Abstract] OR Reluctance[Title/Abstract] OR resistance[Title/Abstract] OR refusal[Title/Abstract] OR acceptance[Title/Abstract] OR willingness[Title/Abstract] OR behavi*[Title/Abstract] OR uptake[Title/Abstract] OR rate*[Title/Abstract] OR recei*[Title/Abstract] OR aware*[Title/Abstract] OR coverage[Title/Abstract] ) AND nurs*[Title/Abstract] AND (barriers[Title/Abstract] OR factor*[Title/Abstract] OR opinion[Title/Abstract] OR determinant[Title/Abstract] OR respons*[Title/Abstract] OR predictor[Title/Abstract] OR intention[Title/Abstract] OR skeptic*[Title/Abstract] OR belief motiv*[Title/Abstract] OR perception*[Title/Abstract] OR intention*[Title/Abstract] OR attitude*[Title/Abstract] OR decision[Title/Abstract] OR belief*[Title/Abstract]) | 1917 |

| **Web of Science (Science citation index expanded)** | |
| --- | --- |
| **Search strategy** | **N. of records** |
| TI=(vaccin*) AND TS=(nurs*) AND TS= (hesitancy OR reluctance OR resistance OR refusal OR acceptance OR willingness OR behavi* OR uptake OR rate* OR recei* OR aware OR coverage) AND TI= (barrier* OR factor* OR opinion* OR determinant* OR respons* OR predictor* OR intention OR skeptic* OR belief motiv* OR perception* OR intention* OR attitude* OR decision OR belief*) | 495 |

| **Scopus** | |
| --- | --- |
| **Search strategy** | **N. of records** |
| TITLE ( vaccin* ) AND TITLE-ABS ( nurs* ) AND TITLE-ABS ( hesitancy OR reluctance OR resistance OR refusal OR acceptance OR willingness OR behavi* OR uptake OR rate* OR recei* OR aware OR coverage ) AND TITLE-ABS ( barrier* OR factor* OR opinion* OR determinant* OR respons* OR predictor* OR intentionOR skeptic* OR belief AND motiv* OR perception* OR intention* OR attitude* OR decision OR belief* ) | 178 |

| **Cinahl** | |
| --- | --- |
| **Search strategy** | **N. of records** |
| AB (MH "Personnel, Health Facility+" OR MH "Nurses+" ) AND (barriers OR factor* OR opinion OR determinant OR respons*OR predictor OR intention OR skeptic* OR belief OR motiv* OR perception*OR intention* OR attitude* OR decision) AND (hesitancy OR reluctance OR resistance OR refusal OR acceptance OR willingness OR behavi* OR uptake OR aware OR rate* OR recei* OR coverage ) AND (MH "Immunization+") | 136 |

| **Eric** | |
| --- | --- |
| **Search strategy** | **N. of records** |
| (Nurs*) AND (barriers OR factor OR opinion OR determinant OR response OR predictor OR intention OR skeptic* OR belie* OR moti* OR perception OR intention OR attitude OR decision) AND (hesitancy OR reluctance OR resistance OR refusal OR acceptance OR willingness OR behaviour* OR uptake OR aware OR rate OR recei* OR coverage) AND (Immunization) | 29 |

| **Joanna briggs Institute** | |
| --- | --- |
| **Search strategy** | **N. of records** |
| (vaccination and nurse and (hesitancy or reluctance or resistance or refusal or acceptance or willingness or behavi* or uptake or rate* or recei* or aware or coverage) and (barrier* or factor* or opinion* or determinant* or respons* or predictor* or intention or skeptic* or belief or motiv* or perception* or intention* or attitude* or decision or belief*)) | 80 |

| **Cochrane database** | |
| --- | --- |
| **Search strategy** | **N. of records** |
| (vaccine OR vaccination):ti,ab,kw AND (hesitancy or reluctance or resistance or refusal or acceptance or willingness or behavi* or uptake or rate* or recei* or aware or coverage):ti,ab,kw AND (nurs*):ti,ab,kw" | 607 |
