# Supplementary figures and images for: Determinants and Motivations of Vaccination Hesitancy and Uptake in Nurses: A Systematic Review and Meta‐Analysis
Source: J Clin Nurs. 2025 Jun 25;34(10):4005–37. doi: 10.1111/jocn.17852 (PMC12409242; doi:10.1111/jocn.17852)

**Figure S1** Forest plot of the prevalence of influenza vaccine uptake among nurses.

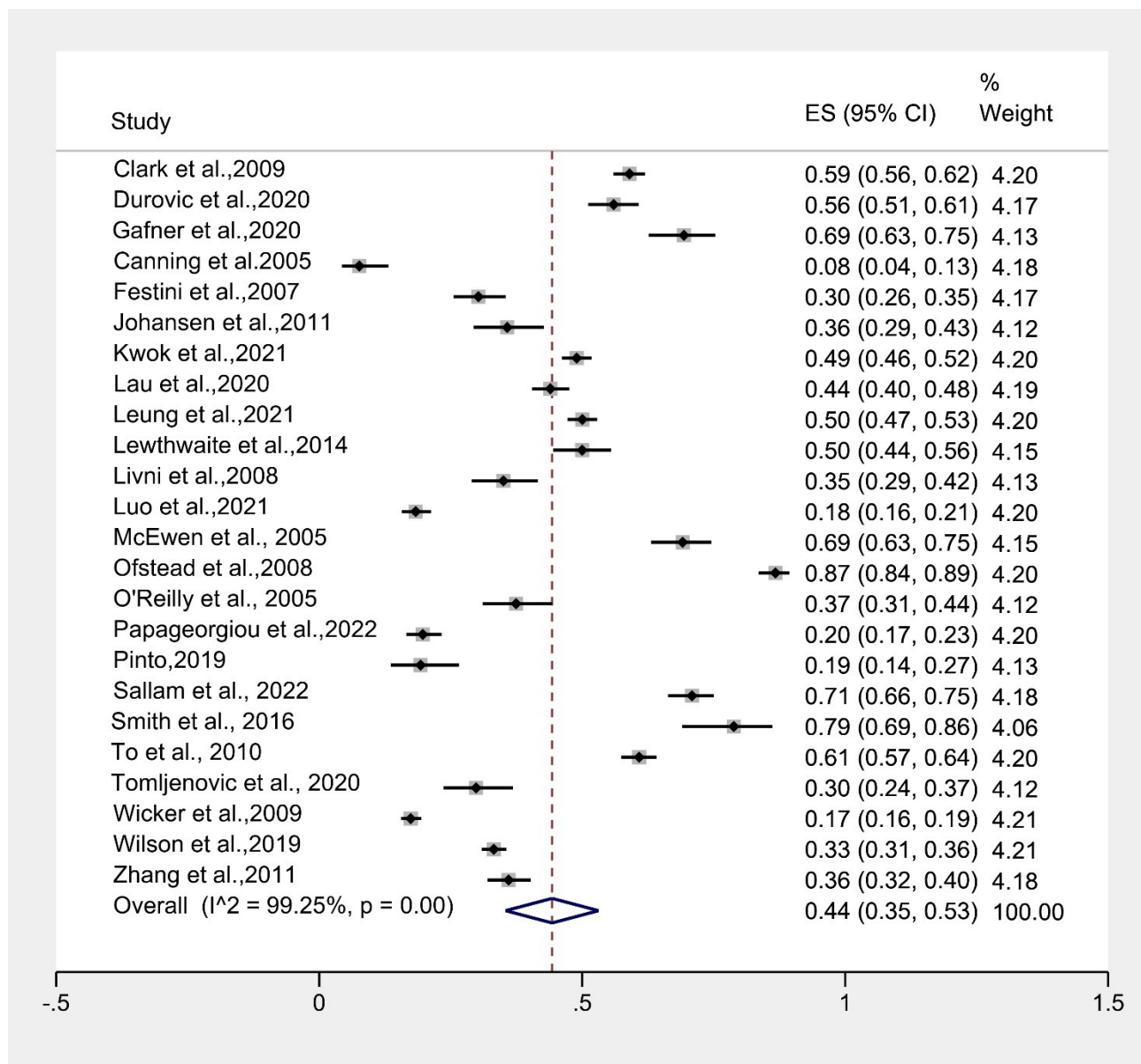

Supplement: Supplementary file 3 — Figure S1 [file JOCN-34-4005-s001.pdf]
